# Further Observations on the Treatment of Chronic Inflammation of the Bladder by Injections of Nitrate of Silver

**Published:** 1849-09

**Authors:** 


*Further Observations on the Treatment of chronic Inflammation of the bladder by Injections of Nitrate of silver, with Cases.*—By Robert L. MacDonnell M. D.—Licentiate of the King and Qeen's College of Physicians, and of the Royal College of Surgeons, Ireland; Physician to the Montreal General Hospital, Lecturer on the Institutes of Medicine, University of M'Gill College.

In a paper, published in the Third Volume of this Journal, I drew the attention of Surgeons, to the great utility of *Injections of nitrate of silver into the bladder, in chronic inflammation of that organ*; and, in support of my views, I adduced some remarkable instances of their succesful employment, which had occurred both in my private and hospital practice. It is with the hope of placing the method of treating a disease, hitherto considered incurable, which one of the most eminent surgeons in the world—Sir Benjamin Brodie—considers the "opprobium of Surgery," and says, "there is no disease for which an approved method of treatment is more wanted," in that position in surgery, which, I feel convinced it deserves to occupy, that I have laid the following case before the profession.

Since my first paper was published, I have cured a great number of persons affected with this disease, but I have selected the following cases from amongst them, because, in them, the cure was *solely, effected by the injections*, whereas, in some of the others, general treatment was likewise employed; and in some, the affection of the bladder, was complicated with organic change of the prostrate gland, with strictures of the urethra, and, in one instance, with urinary fistulæ and stricture—complications requiring special treatment, and which, some might suppose, assisted in relieving the affection of the bladder; although, I am quite satisfied, the cures of the vesical inflamation were due to the injections alone. I have also omitted some mild cases of the disease, because, as stated in my former paper, they might have been cured by remedies, generally known to surgeons, and therefore, are not so valuable, as evidence in favor of the method by injections. But, my principal reason for selecting the following examples is, that we have in them, unquestionable testimony of the utility of the practice I advocate—for in all general treament and the usual remedies *had been carefully and perseveringly employed, without success; and in one, the age af the patient, and the duration of the disease, were most unfavourable for testing the merits of the treatment, yet in all, the cure was complets and permanent.*

Case 1.Mr.—,aged 33, had several attacks of gonorrhœa which had been cured in the usual manner, and had caused him very little anxiety, except the last one, which was contracted in April, 1848, and was soon followed by symptoms indicating inflamation of the bladder. For the latter affection he had been under the care of a surgeon in this city, from May till September 27th, when he consulted me. He then complained of being obliged to make water almost every ten or fifteen minutes during the day, and between twenty and thirty minutes during the night, accompanied by pain and heat about the region of the bladder, scalding along the urethra particularly as the last drops were passing. The urine was usually expelled in a jet, and, when allowed to remain at rest, it threw down a copious deposit of pus and blood, and some flakes of lymph; no discharge from the urethra. He had lost flesh and strength, and had become dispirited and extremely irritable, and his countenance was haggard and anxious. Tongue clean,—appetite good.—bowels regular,—pulse 80, small and weak,—no headache. The sleep being so frequently disturbed by necessity of emptying the bladder, he rises in the morning languid and exhausted. In order to ascertain the condition of the urethra, a No. 11 (Weiss) bougie was passed, and met with no obstruction, nor was any pain complained of, except as it was passing over the neck of the bladder. The deposit thrown down by the urine was examined under the microscope, and found to be composed of pus and blood globules, epithelial scales, and some crystals of triple phosphate. He was ordered Dover's powder at night.

Sept. 28, 6 o'clock, A .M.—He states that since yesterday he passed water about fifty times. At 6 o'clock P. M . I injected the bladder with a solution of nitrate of silver containing two grains to the ounce, which caused very little pain. Ordered him to take Dover's powder at night, a warm bath immediately, and drink plenty of weak tea.

Sept. 29, 1 o'clock P. M.—Pain ceased immediately on entering the bath. He has passed water only 4 times since 6 o'clock yesterday and the evacuation of the bladder is not accompanied by pain or scalding, and the pain above the pubis in the perinæum has completely disappeared. In all respects he feels much better, and the countenance has lost the haggard appearance it had latt rly assumed. The urine is now clear and devoid of sediment. Barley-water for drink, and warm bath at bed time. Six o'clock, P. M. Passed water only once since last report, and even then he did so from a feeling that it was not right to allow the bladder to remain distended, and not from a desire to empty it.

Sept.30—Passed water only once between ten o'clock last night and eight this morning.

Oct.1.—Within the last twenty-four hours has made water only three times

Oct. 2.—Injected the bladder again to-day with a solution of the same strength, which gave scarcely any uneasiness.

Oct. 6.—No return of the malady. He can now keep his water for six or seven hours, and it is quite clear and free from any pus or blood globules when examined under the microscope.

I have seen this gentleman very lately, and he assured me that he has not had the least return of his complaint, although he has imprudently exposed himself to wet, and severe cold on many occasions since I ceased to attend him.

The following case has been transmitted to me by Dr. Shewbridge Connor, Physician to the fever Hospital, Carlow, one of the most eminet practitioners of Ireland, whose testimony must be considered as highly valuable.

"Case II.—I attended Mr.——, a respectable farmer some miles from this, whilst he was laboring under fever, complicated with bronchitis.—When convalescent, he informed me that "for years he was obliged to empty his bladder oftener than any one else. He could not drive a mile without stopping several times; he said that occasionally he passed whitish-looking matter. Warm baths, buchu, and other usual remedies were prescribed by me without much effect, perhaps partly in consequence of his presisting to superintend his farm-work in cold damp weather.

In October, late one evening, he sent for me, and begged me to bring something to relieve him, as he was obliged to be up every minute, and was suffering intensely all the time. Fortunately, I had read some days previously your paper (*Dublin Medical Press*, Oct. 6, 1847) on "Injections of nitrate of silver in Chronic Inflammation of the Bladder." No practitioner that I have met, had or has tried it, but aware of the power of the medicine inother inflammations, I had no hesitation in acting on your suggestion, and accordingly injected five grains of nitrate of silver, two drachms of tincture of hyoscyamus, and four ounces of distilled water. The instrument was clumsy and not suitable,—a small brass enema syringe, connected by a piece of bladder with the end of a gum elastic catheter. At the moment I could not get a glass syringe. Taking care that the nitrate of silver should remain only a moment in the syringe, I injected it, and compressed the catheter for about a minute or so to prevent the patient instantly discharging it, which he had a great desire to do. I then withdrew the catheter and left him for the night, having ordered (needlessly perhaps) flannels wrung out of hot water, to be applied for some time to the pubic region. Next day, he informed me that he had slept well, and had no occasion to get up for six hours. I passed his door and meet him once a week at least, and he has never mentioned the subject unless when asked about it, though, at times, he feels a return of his complaint, which is so trifling, however, that he does not like *troubling* the Doctor. The long-suffering of the farmer class, when a Doctor is to be consulted, is the most remarkable in this part of the world.

"Mr.——now travels far by railway—not a verypleasant conveyance for a man with irritable bladder."

Shewbridge Connor, M. D.,
Co. Fever Hospital, Carlow,

CASE III.—Mr.——, aged 64, stout plethoric habit, contracted gonorrhœa about thirty years ago, and since then has suffered from the following symptoms:—Pain across the urethra, after sexual intercourse, and after passing water,—great pain over the region of the bladder and in the perinæum,—urine passed every half hour, and sometimes much oftener.—The urine was always fœtid, turbid, and threw down a copious deposit of pus, blood, flakes of lymph, and mucus. At various times, his sufferings have been so great as to keep him confined to bed for months, and he has frequently been attacked with spasmodic stricture, causing retention of urine. According to his own statement, he has consulted medical men in almost every city in North America; for, being the proprietor of a public exhibition, he has visited the principal cities frequently during the last nine or ten years. He has also passed through the hands of numerous quacks and charlatans. He appears to have derived most benefit from the services of a surgeon in Richmond, N. Y., who advised him to use capulses of balsam of copaiba. which he thinks have kept the disease in abeyance, more than any other treatment. In 1846, he applied to a surgeon in this city who gave him buchu and other remedies without any benefit. Between 1846 and 1848 he consulted some eminent practitioners in Philadelphia, but deriving no relief from their remedies, he began, as he says, "to doctor himself with medicine similar to what he got in Richmond, which gave him temporary relief."

Sept. 12, 1848.—He consulted me, and in addition to the foregoing he complained of pain when he sat down suddenly, but had none on going to stool. The pain on pressure over the bladder was very great, but he had no pain shooting along the course of the ureter or to the kidneys; never passed any calculi. A No. 9 (Weiss) bougie was introduced into the bladder without any difficulty, except near the neck of the bladder, where the passage of it caused pain, No.10 and No. 11 passed with equal ease.—The urine voided during the visit, was examined, and found to contain a large deposit of pus and blood globules, flakes of epithelium and crystals of triple phosphate, and, on being tested, the supernatant fluid was found to be highly albuminos. It was also much more fœtid than I have found the recent evacuation of urine to be—even in a similar cases. He was ordered to take that night, a draught composed of spirits of camphor, sweet spirits of nitre, and tincture of hysoscyamus, and the next morning the bladder was injected with a solution of nitrate, of silver, two grains to theounce; and he was advised to take a warm bath and to drink plenty of barley-water.

Sept. 14.—For a few hours after the injection, he was obliged to empty the bladder every half hour—but towards morning, he could retain his urine for two hours and half at a time. The smarting pain in the region of the bladder is much relieved; no pain over the pubis or in the perinæum. Continue medicines.

Sept. 15.—Last night, the weather becoming suddenly very cold, he made water more frequently than he had done during the day, but it was quite free from odour, and presented a healthy appearance. Continue medicines.

Sept. 20.—Injected the bladder again to-day, with a solution of four grains to the ounce.

Sept. 25.—Injected the bladder again, with a solution of the same strength as the last.

Sept. 30.—Injected solution, five grains to the ounce.

Oct. 3.—Repeated the injection. He can now retain his water for six hours at a time, and is quite free from offensive odour, and clear.

Oct. 6.—Injected again.

Oct. 9.—Injected a solution of the same strength. He can now retain his urine for seven or eight hours at a time, and, in short, feels no inconvenience from his old complaint.

This gentleman, who had passed several years in a warm climate, spent all last winter in Montreal, which was one of the most severe and coldest that has been for several years, yet he went out almost every day, and did not experience the least relapse, nor does he now, May 20, suffer from the least symptom of the excruciating and exhausting disease he labored under, for so many years, and which he had believed to be perfectly incurable.

Case IV.— r.———, aged 30, in the active practice of his profession, in the Eastern Townships, consulted me for several attack of chronic cystitis which he had ineffectually attempted to cure by the usual remedies, and for which he had been under the treatment of a physician in this city, for nearly three months, without deriving much benefit. He stated that having been exposed to severe cold and wet, during a long drive in the autumn, he remarked, on reaching home, that he had some pain in making water, and scalding along the urethra. Of this he took little notice at the time, but the same symptoms continued unrelieved by the remedies he employed, and were soon attended with an urgent desire to empty the bladder almost every hour; the urine was passed in jets, and of turbid whitish color, throwing down a copious deposit of pus and blood when it had lain in repose for a short time, and he was affected with severe pain over the region of the bladder and in the perinæum at times which amounted to agony when riding on horseback, in the performance of his professional duties. He had latterly begun to loose flesh; and irritability of the mucus membrane of the intestinal canal ,marked by frequently returning attacks of diarrhœa, added much to his sufferings. When he consulted me, he was much emaciated, the countenance wore a haggard and anxious expression; the pulse was small and quick; skin harh and dry, tongue, red, chapped; appetite bad, vomiting frequently, scrcely any food remaining on his stomach, except oatmeal porridge, bowels sometimes confined, but more frequently loose; sleep greatly disturbed by the necessity of frequent emptying the bladder; and his spirits, which before were good, were low and desponding. He was obliged to pass water almost every half hour, and when examined under the microscope, the deposit presented precisely the same appearance that were discovered in the foregoing cases.

I ordered him a combination of mercury with chalk, rhubarb, extract of henbane, and acetate of morphia, all in small doses, to allay the intestinal irritation, and four ounces of distilled water, holding in solution eight grains of nitrate of silver, were injected into the bladder; and he was advised to take a warm bath immediately after the operation. The next day he felt much better, and the improvement continuing, he was not obliged to have the injection repeated. I again saw him last January, nine months after the operation, when he appeared much improved, had gained flesh and strength, and had not the least return of his former malady. I had written to him a few days before his arrival in town, and in reply, I received the following note—

January 10, 1849.

MY DEAR DOCTOR,—Your note was received, but not so soon as it should have been, owing to some neglect of the Post Office. I am happy to comply with your request, to furnish replies to your queries, as to my own case of cystitis. The disease has not returned, nor has it troubled me in the least, since I recovered from the first attack. I did not feel any inconvenience from the injection of the nitrate of silver into the bladder. I am happy to say, I never witnessed a more perfect cure than in my own case.

I remain my Dear Doctor,
Yours, &c.

R. L. M'Donnell, M. D. * * *

As the foregoing cases may meet the eye of some practitioner who has not seen my former paper on this subject, I shall make no apology for introducing here the directions laid down in it for injecting the bladder:—"The patient being placed either in the erect position or on a sofa, a gum elastic catheter, about the size of No. 9 or 10 (Weiss) is introduced, and water at the temperature of 98 deg. Fahr., is injected through this into the bladder, by means of a caoutchouc bag, or what I prefer, a syringe, with a "three-way valve," by which the fluid can be drawn back from the cavity if necessary. After the bladder has been completely cleansed of any fœtid urine and mucus which may be contained in it, the solution of the caustic, being heated to the same degree, is to be introduced in a similar manner, and allowed to remain there for about one minute, care being taken, by compressing the urethra, to prevent its being forcibly ejected by the violent straining that is certain to be induced. The quantity of water or solution should never exceed four ounces, for though the bladder in its healthy state is capable of containing nearly a pint and a half of urine, without being over distended, yet as the quantity it is capable of retaining in severe chronic inflammation seldom exceeds a few tablespoonfuls, the bladder accommodates itself to its diminished contents, and gradually becomes smaller, and consequently a large injection would act injuriously in two ways—by over-distending the organ, or by passing up into the ureters. In fact, we find it unnecessary to use a larger quantity of the solution than I have mentioned, for it requires some address to use even that amount without resorting to force. The patient is then ordered a warm bath, and should the urine become bloody or mixed with shreddy concretions, he should use frequent fomentations and anodynes. But these symptoms seldom last more than a few hours, and our patients should always be informed that such consequences are likely to be the immediate effects of the operation."

The strength of the injection has seldom to be increased beyond five grains to the ounce, although in one instance, that of an old gentleman, aged seventy-two, I had to increase the strength *gradually* to ten grains to the ounce before a satisfactory effect was produced. It is, however, always better to commence with a weak solution, which may be made stronger, according to the circumstances of each case, and the judgment of the practitioner. Some of my patients have hesitated about undergoing treatment by injections, in consequence of their advanced age, but though the disease is not in such cases so easily cured, as in the young subject, it is still in the great majority of instances remediable by the same means, as was proved by the great relief obtained by a patient aged *seventy-six*, who was under my care in the Montreal General Hospital, within the last month, into whose bladder I injected, on two occasions, a solution of nitrate of silver, two grains to the ounce. He left the Hospital of his own accord, May 23, quite free from his former complaint.

The Surgeon should, in fact, show his patient that all general treatment and local and general remedies having failed, he has only two alternatives to choose between—a life of misery and suffering, a burthen to himself, and incapable for the enjoyment of society, or the performance of business—and submission to a plan of treatment which has been eminently successful in cases equally protracted and aggravated as his own, and in patients equally old and infirm, and who like him had spent time and money, and exhausted their patience in ineffectual efforts to get rid of a disease so formidable, so excruciating, and so disgusting to themselves and others, as Chronic Inflammation of the Bladder.

Montreal, May, 1849.—*British American Journal.*

